# (Ethyl­enediamine-κ^2^ *N*,*N*′)bis­[3-(2-pyridyl)-5-(4-pyrid­yl)-1,2,4-triazolato-κ^2^ *N* ^2^,*N* ^3^]zinc(II) methanol solvate dihydrate

**DOI:** 10.1107/S1600536809001688

**Published:** 2009-01-17

**Authors:** Lin Cheng, Yan-Yan Sun, Ya-Wen Zhang, Jian-Quan Wang

**Affiliations:** a Department of Chemistry and Chemical Engineering, Southeast University, Nanjing 211189, People’s Republic of China; b Department of Chemistry and Chemical Engineering, State Key Laboratory of Coordination Chemistry, Nanjing University, Nanjing 211189, People’s Republic of China

## Abstract

The asymmetric unit of the title compound, [Zn(C_12_H_8_N_5_)_2_(C_2_H_8_N_2_)]·CH_3_OH·2H_2_O, contains a Zn^II^ cation, octahedrally coordinated by two 3-(2-pyrid­yl)-5-(4-pyrid­yl)-1,2,4-triazolate anions, a chelating ethane-1,2-diamine mol­ecule, a methanol solvent mol­ecule, and two crystal water mol­ecules. In the crystal packing, complex mol­ecules are linked by hydrogen bonds into a two-dimensional layer.

## Related literature

For related structures, see: Wang *et al.* (2005 ▶). For general background, see: Kesanli & Lin (2003 ▶).
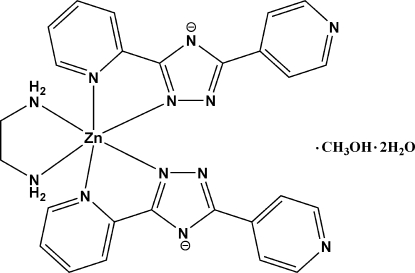


## Experimental

### 

#### Crystal data

[Zn(C_12_H_8_N_5_)_2_(C_2_H_8_N_2_)]·CH_4_O·2H_2_O
*M* *_r_* = 638.02
Triclinic, 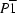

*a* = 8.3323 (8) Å
*b* = 13.0268 (12) Å
*c* = 14.9444 (14) Å
α = 66.404 (2)°
β = 79.751 (2)°
γ = 81.437 (2)°
*V* = 1457.3 (2) Å^3^
*Z* = 2
Mo *K*α radiation
μ = 0.90 mm^−1^
*T* = 293 (2) K
0.25 × 0.22 × 0.16 mm

#### Data collection

Bruker APEX CCD diffractometer
Absorption correction: multi-scan (*SADABS*; Sheldrick, 2000 ▶) *T*_min_ = 0.807, *T*_max_ = 0.870
11395 measured reflections
5643 independent reflections
4532 reflections with *I* > 2σ(*I*)
*R*_int_ = 0.030

#### Refinement

*R*[*F* ^2^ > 2σ(*F* ^2^)] = 0.055
*wR*(*F* ^2^) = 0.130
*S* = 1.04
5643 reflections
388 parameters
H-atom parameters constrained
Δρ_max_ = 0.61 e Å^−3^
Δρ_min_ = −0.56 e Å^−3^

### 

Data collection: *SMART* (Bruker, 2000 ▶); cell refinement: *SAINT* (Bruker, 2000 ▶); data reduction: *SAINT*; program(s) used to solve structure: *SHELXTL* (Sheldrick, 2008 ▶); program(s) used to refine structure: *SHELXTL*; molecular graphics: *SHELXTL*; software used to prepare material for publication: *SHELXTL*.

## Supplementary Material

Crystal structure: contains datablocks I, global. DOI: 10.1107/S1600536809001688/kp2199sup1.cif

Structure factors: contains datablocks I. DOI: 10.1107/S1600536809001688/kp2199Isup2.hkl

Additional supplementary materials:  crystallographic information; 3D view; checkCIF report

## Figures and Tables

**Table 1:** Selected bond lengths (Å)

Zn1—N7	2.096 (3)
Zn1—N2	2.103 (2)
Zn1—N11	2.133 (3)
Zn1—N12	2.148 (3)
Zn1—N1	2.269 (3)
Zn1—N6	2.293 (3)

**Table 2:** Hydrogen-bond geometry (Å, °)

*D*—H⋯*A*	*D*—H	H⋯*A*	*D*⋯*A*	*D*—H⋯*A*
N11—H11*B*⋯O1*W*^i^	0.90	2.27	3.166 (4)	172
N11—H11*C*⋯O2*W*^ii^	0.90	2.17	3.034 (4)	161
N12—H12*B*⋯N5^iii^	0.90	2.54	3.321 (4)	146
N12—H12*C*⋯O1*W*	0.90	2.45	3.331 (4)	165
O1—H1*B*⋯N4^iv^	0.90	2.02	2.897 (4)	163
O1*W*—H1*WA*⋯N3^v^	0.85	2.04	2.887 (4)	171
O1*W*—H1*WB*⋯N8	0.85	2.09	2.859 (4)	150
O2*W*—H2*WA*⋯O1	0.85	1.94	2.778 (4)	169
O2*W*—H2*WB*⋯O1*W*	0.85	2.00	2.787 (4)	153

Symmetry codes: (i) 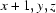; (ii) 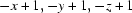; (iii) 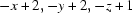; (iv) 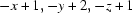; (v) 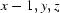.

**Fig. 1.**
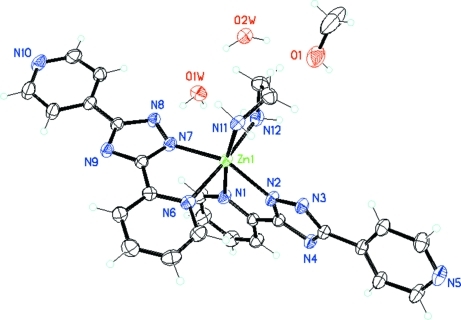
Coordination environment of ZnII with the 30% displacement ellipsoids.

**Fig. 2.**
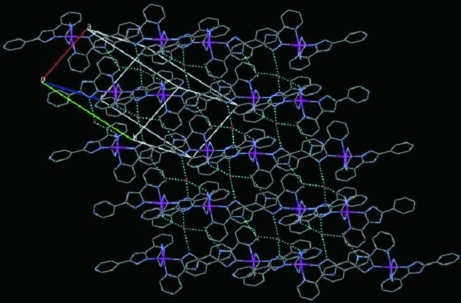
The two-dimensional hydrogen bond network of the title compound.

## supplementary crystallographic information

### Comment

Recently, there has been significant interest in the rational design and synthesis of chiral coordination polymers due to their potential functions, such as enantioselective separations and catalysis, which could be obtained by using chiral and achiral ligands (Kesanli *et al.* 2003; Wang *et al.* 2005). The use of achiral ligands is more attractive since usually such ligands are easier to be generated. Asymmetric ligands, as achiral ligands, have been widely used to construct those non-centrosymmetrical polymers. Unfortunately, here, we synthesed a centrosymmetrical complex Zn(C_12_H_8_N_5_)_2_(C_2_H_8_N_2_).CH_3_OH.2H_2_O, with an asymmetric 3-(2-pyridyl)-5-(4-pyridyl)-4_H_-1,2,4-triazole ligand.

The asymmetric unit of the title compound, contains one Zn^II^ cation, two 3-(2-pyridyl)-5-(4-pyridyl)-1,2,4-triazolato anions, one chelating ethane-1,2-diamine, methanol solvate and two crystal water molecules. The complex is mononuclear with Zn^II^ in a distorted octahedral geometry of two chelating triazolato anions, and one chelating ethane-1,2-diamine ligand. Each triazolato group acts as a bidentate chelating ligand whereas the N atom of 4-pyridyl group does not take part in coordination (Table 1and Fig. 1). In the crystal, the complex molecules are linked by N—H···O, O—H···N and O—H···O hydrogen bonds into a two-dimensional layer (Table 2 and Fig. 2).

### Experimental

A mixture of 3-(2-pyridyl)-5-(4-pyridyl)-4_H_-1,2,4-triazole (0.0446 g, 0.2 mmol), ZnSO_4_.7 H_2_O (0.0288 g, 0.1 mmol), ethane-1,2-diamine (0.1 ml), methanol (2 ml) and water (2 ml) was stirred for 0.5 h at room temperature, and then filtered. The filtrate was allowed to evaporate slowly at room temperature. After 2 weeks, colourless needle crystals were obtained in 34% yield (0.0217 g) based on Zn^II^.

### Refinement

H atoms bonded to N and O atoms were located in a difference map with the distances of N—H = 0.90 and O—H = 0.85–0.90 Å. Other H atoms were positioned geometrically and refined using a riding model with C—H = 0.93–0.97 Å and with *U*_iso_(H) = 1.2.

### Crystal data

**Table:**

[Zn(C_12_H_8_N_5_)_2_(C_2_H_8_N_2_)]·CH_3_O·2H_2_O	*Z* = 2
*M**_r_* = 638.02	*F*(000) = 664
Triclinic, *P*1	*D*_x_ = 1.454 Mg m^−^^3^
Hall symbol: -P 1	Mo *K*α radiation, λ = 0.71073 Å
*a* = 8.3323 (8) Å	Cell parameters from 783 reflections
*b* = 13.0268 (12) Å	θ = 2.5–28.0°
*c* = 14.9444 (14) Å	µ = 0.90 mm^−^^1^
α = 66.404 (2)°	*T* = 293 K
β = 79.751 (2)°	Needle, colourless
γ = 81.437 (2)°	0.25 × 0.22 × 0.16 mm
*V* = 1457.3 (2) Å^3^	

### Data collection

**Table:**

Bruker APEX CCD diffractometer	5643 independent reflections
Radiation source: fine-focus sealed tube	4532 reflections with *I* > 2σ(*I*)
graphite	*R*_int_ = 0.030
φ and ω scans	θ_max_ = 26.0°, θ_min_ = 1.8°
Absorption correction: multi-scan (*SADABS*; Sheldrick, 2000)	*h* = −10→10
*T*_min_ = 0.807, *T*_max_ = 0.870	*k* = −16→16
11395 measured reflections	*l* = −18→18

### Refinement

**Table:**

Refinement on *F*^2^	Primary atom site location: structure-invariant direct methods
Least-squares matrix: full	Secondary atom site location: difference Fourier map
*R*[*F*^2^ > 2σ(*F*^2^)] = 0.055	Hydrogen site location: inferred from neighbouring sites
*wR*(*F*^2^) = 0.130	H-atom parameters constrained
*S* = 1.04	*w* = 1/[σ^2^(*F*_o_^2^) + (0.0579*P*)^2^ + 0.853*P*] where *P* = (*F*_o_^2^ + 2*F*_c_^2^)/3
5643 reflections	(Δ/σ)_max_ < 0.001
388 parameters	Δρ_max_ = 0.61 e Å^−^^3^
0 restraints	Δρ_min_ = −0.56 e Å^−^^3^

### Special details

**Table:**

Geometry. All e.s.d.'s (except the e.s.d. in the dihedral angle between two l.s. planes) are estimated using the full covariance matrix. The cell e.s.d.'s are taken into account individually in the estimation of e.s.d.'s in distances, angles and torsion angles; correlations between e.s.d.'s in cell parameters are only used when they are defined by crystal symmetry. An approximate (isotropic) treatment of cell e.s.d.'s is used for estimating e.s.d.'s involving l.s. planes.
Refinement. Refinement of *F*^2^ against ALL reflections. The weighted *R*-factor *wR* and goodness of fit *S* are based on *F*^2^, conventional *R*-factors *R* are based on *F*, with *F* set to zero for negative *F*^2^. The threshold expression of *F*^2^ > σ(*F*^2^) is used only for calculating *R*-factors(gt) *etc*. and is not relevant to the choice of reflections for refinement. *R*-factors based on *F*^2^ are statistically about twice as large as those based on *F*, and *R*- factors based on ALL data will be even larger.

### Fractional atomic coordinates and isotropic or equivalent isotropic displacement parameters (Å^2^)

**Table:**

	*x*	*y*	*z*	*U*_iso_*/*U*_eq_	
Zn1	0.71857 (5)	0.78434 (3)	0.29062 (3)	0.03871 (14)	
C1	0.4382 (5)	0.9641 (3)	0.1713 (3)	0.0551 (10)	
H1A	0.4027	0.9023	0.1664	0.066*	
C2	0.3536 (5)	1.0670 (4)	0.1313 (3)	0.0636 (11)	
H2A	0.2637	1.0748	0.0993	0.076*	
C3	0.4042 (5)	1.1584 (3)	0.1393 (3)	0.0609 (11)	
H3A	0.3492	1.2293	0.1127	0.073*	
C4	0.5377 (4)	1.1433 (3)	0.1875 (3)	0.0477 (9)	
H4A	0.5729	1.2036	0.1949	0.057*	
C5	0.6183 (4)	1.0380 (3)	0.2245 (2)	0.0357 (7)	
C6	0.7672 (4)	1.0124 (3)	0.2716 (2)	0.0350 (7)	
C7	0.9746 (4)	1.0178 (3)	0.3288 (2)	0.0364 (7)	
C8	1.0995 (4)	1.0544 (3)	0.3656 (2)	0.0378 (7)	
C9	1.1963 (4)	0.9778 (3)	0.4322 (3)	0.0456 (8)	
H9A	1.1866	0.9012	0.4534	0.055*	
C10	1.3081 (4)	1.0159 (3)	0.4672 (3)	0.0549 (10)	
H10A	1.3723	0.9627	0.5123	0.066*	
C11	1.2366 (5)	1.1968 (3)	0.3747 (3)	0.0597 (11)	
H11A	1.2510	1.2728	0.3531	0.072*	
C12	1.1207 (5)	1.1674 (3)	0.3365 (3)	0.0512 (9)	
H12A	1.0575	1.2223	0.2918	0.061*	
C13	1.0318 (4)	0.8471 (3)	0.1141 (3)	0.0522 (9)	
H13A	1.0659	0.8844	0.1482	0.063*	
C14	1.1280 (5)	0.8449 (3)	0.0311 (3)	0.0601 (11)	
H14A	1.2243	0.8807	0.0088	0.072*	
C15	1.0810 (5)	0.7890 (3)	−0.0194 (3)	0.0552 (10)	
H15A	1.1451	0.7859	−0.0761	0.066*	
C16	0.9368 (4)	0.7374 (3)	0.0155 (3)	0.0447 (8)	
H16A	0.9026	0.6980	−0.0166	0.054*	
C17	0.8445 (4)	0.7457 (3)	0.0990 (2)	0.0372 (7)	
C18	0.6844 (4)	0.6996 (3)	0.1399 (2)	0.0360 (7)	
C19	0.4638 (4)	0.6307 (3)	0.1630 (2)	0.0370 (7)	
C20	0.3338 (4)	0.5731 (3)	0.1523 (3)	0.0389 (8)	
C21	0.2038 (4)	0.5364 (3)	0.2252 (3)	0.0484 (9)	
H21A	0.1938	0.5495	0.2828	0.058*	
C22	0.0886 (5)	0.4799 (3)	0.2112 (3)	0.0583 (10)	
H22A	0.0012	0.4566	0.2608	0.070*	
C23	0.2181 (5)	0.4939 (4)	0.0620 (3)	0.0633 (11)	
H23A	0.2245	0.4800	0.0051	0.076*	
C24	0.3379 (5)	0.5521 (3)	0.0684 (3)	0.0527 (10)	
H24A	0.4212	0.5771	0.0164	0.063*	
C25	0.8006 (5)	0.6203 (3)	0.4815 (3)	0.0540 (10)	
H25A	0.8431	0.6727	0.5008	0.065*	
H25B	0.8377	0.5446	0.5230	0.065*	
C26	0.6162 (5)	0.6348 (3)	0.4945 (3)	0.0562 (10)	
H26A	0.5734	0.5796	0.4789	0.067*	
H26B	0.5750	0.6239	0.5623	0.067*	
N1	0.5690 (3)	0.9484 (2)	0.2168 (2)	0.0425 (7)	
N2	0.8401 (3)	0.9090 (2)	0.3005 (2)	0.0379 (6)	
N3	0.9767 (3)	0.9114 (2)	0.3379 (2)	0.0391 (6)	
N4	0.8461 (3)	1.0852 (2)	0.2877 (2)	0.0384 (6)	
N5	1.3297 (4)	1.1242 (3)	0.4403 (3)	0.0584 (9)	
N6	0.8917 (3)	0.7990 (2)	0.1493 (2)	0.0425 (7)	
N7	0.6034 (3)	0.7089 (2)	0.2217 (2)	0.0392 (6)	
N8	0.4580 (3)	0.6639 (2)	0.2376 (2)	0.0405 (6)	
N9	0.6030 (3)	0.6513 (2)	0.0994 (2)	0.0411 (7)	
N10	0.0943 (4)	0.4566 (3)	0.1321 (3)	0.0618 (9)	
N11	0.8616 (3)	0.6409 (2)	0.3777 (2)	0.0418 (7)	
H11B	0.9644	0.6557	0.3761	0.050*	
H11C	0.8363	0.5759	0.3779	0.050*	
N12	0.5630 (3)	0.7487 (2)	0.4287 (2)	0.0466 (7)	
H12B	0.5435	0.7894	0.4666	0.056*	
H12C	0.4597	0.7511	0.4173	0.056*	
C27	0.3055 (11)	0.6293 (5)	0.7695 (7)	0.204 (5)	
H27A	0.2189	0.6451	0.8151	0.245*	
H27B	0.4091	0.6254	0.7908	0.245*	
H27C	0.2926	0.5587	0.7666	0.245*	
O1	0.2993 (5)	0.7199 (3)	0.6702 (3)	0.1036 (12)	
H1B	0.2698	0.7879	0.6733	0.124*	
O1W	0.2078 (3)	0.7151 (2)	0.37517 (18)	0.0555 (7)	
H1WA	0.1489	0.7772	0.3615	0.067*	
H1WB	0.2587	0.7179	0.3197	0.067*	
O2W	0.1847 (4)	0.6019 (2)	0.5789 (2)	0.0810 (10)	
H2WA	0.2278	0.6300	0.6105	0.097*	
H2WB	0.1918	0.6143	0.5181	0.097*	

### Atomic displacement parameters (Å^2^)

**Table:**

	*U*^11^	*U*^22^	*U*^33^	*U*^12^	*U*^13^	*U*^23^
Zn1	0.0371 (2)	0.0371 (2)	0.0476 (2)	−0.00668 (16)	−0.01032 (17)	−0.01898 (18)
C1	0.052 (2)	0.050 (2)	0.069 (3)	−0.0071 (18)	−0.028 (2)	−0.020 (2)
C2	0.054 (2)	0.061 (3)	0.076 (3)	−0.001 (2)	−0.039 (2)	−0.016 (2)
C3	0.053 (2)	0.050 (2)	0.074 (3)	0.0077 (19)	−0.027 (2)	−0.014 (2)
C4	0.046 (2)	0.039 (2)	0.059 (2)	−0.0010 (16)	−0.0134 (18)	−0.0178 (17)
C5	0.0318 (17)	0.0397 (18)	0.0380 (17)	−0.0050 (14)	−0.0062 (14)	−0.0158 (15)
C6	0.0333 (17)	0.0350 (18)	0.0391 (17)	−0.0033 (14)	−0.0081 (14)	−0.0149 (14)
C7	0.0344 (18)	0.0375 (18)	0.0420 (18)	−0.0040 (14)	−0.0065 (14)	−0.0192 (15)
C8	0.0310 (17)	0.0443 (19)	0.0476 (19)	−0.0059 (14)	−0.0057 (15)	−0.0262 (16)
C9	0.0368 (19)	0.047 (2)	0.061 (2)	−0.0030 (16)	−0.0128 (17)	−0.0254 (18)
C10	0.042 (2)	0.064 (3)	0.068 (3)	0.0016 (19)	−0.0218 (19)	−0.031 (2)
C11	0.060 (3)	0.051 (2)	0.083 (3)	−0.013 (2)	−0.019 (2)	−0.035 (2)
C12	0.053 (2)	0.043 (2)	0.067 (2)	−0.0042 (17)	−0.0212 (19)	−0.0254 (19)
C13	0.044 (2)	0.052 (2)	0.064 (2)	−0.0126 (18)	−0.0050 (19)	−0.024 (2)
C14	0.041 (2)	0.063 (3)	0.070 (3)	−0.0196 (19)	0.003 (2)	−0.019 (2)
C15	0.043 (2)	0.066 (3)	0.055 (2)	−0.0031 (19)	0.0010 (18)	−0.025 (2)
C16	0.0366 (19)	0.053 (2)	0.047 (2)	−0.0043 (16)	−0.0047 (15)	−0.0214 (17)
C17	0.0324 (17)	0.0382 (18)	0.0387 (17)	−0.0017 (14)	−0.0086 (14)	−0.0112 (15)
C18	0.0346 (17)	0.0354 (18)	0.0393 (17)	−0.0014 (14)	−0.0034 (14)	−0.0168 (15)
C19	0.0341 (18)	0.0350 (18)	0.0436 (18)	−0.0027 (14)	−0.0032 (15)	−0.0177 (15)
C20	0.0337 (18)	0.0342 (18)	0.052 (2)	0.0006 (14)	−0.0074 (15)	−0.0208 (16)
C21	0.045 (2)	0.053 (2)	0.052 (2)	−0.0113 (17)	−0.0020 (17)	−0.0247 (18)
C22	0.047 (2)	0.058 (2)	0.070 (3)	−0.0210 (19)	0.003 (2)	−0.023 (2)
C23	0.064 (3)	0.069 (3)	0.076 (3)	−0.016 (2)	−0.006 (2)	−0.046 (2)
C24	0.046 (2)	0.062 (2)	0.064 (2)	−0.0148 (19)	0.0027 (18)	−0.039 (2)
C25	0.055 (2)	0.054 (2)	0.050 (2)	0.0056 (19)	−0.0161 (18)	−0.0174 (19)
C26	0.054 (2)	0.059 (3)	0.053 (2)	−0.014 (2)	0.0035 (19)	−0.019 (2)
N1	0.0390 (16)	0.0405 (16)	0.0522 (17)	−0.0042 (13)	−0.0187 (13)	−0.0166 (14)
N2	0.0332 (15)	0.0340 (15)	0.0532 (17)	−0.0018 (12)	−0.0155 (13)	−0.0199 (13)
N3	0.0335 (15)	0.0385 (16)	0.0516 (17)	−0.0014 (12)	−0.0130 (13)	−0.0212 (13)
N4	0.0373 (15)	0.0340 (15)	0.0491 (16)	−0.0019 (12)	−0.0140 (13)	−0.0181 (13)
N5	0.0494 (19)	0.066 (2)	0.076 (2)	−0.0097 (17)	−0.0190 (17)	−0.0386 (19)
N6	0.0380 (16)	0.0419 (16)	0.0487 (17)	−0.0085 (13)	−0.0056 (13)	−0.0169 (14)
N7	0.0336 (15)	0.0425 (16)	0.0463 (16)	−0.0072 (12)	−0.0037 (12)	−0.0213 (13)
N8	0.0341 (15)	0.0464 (17)	0.0470 (16)	−0.0084 (13)	0.0008 (12)	−0.0252 (14)
N9	0.0357 (15)	0.0474 (17)	0.0475 (16)	−0.0072 (13)	−0.0029 (13)	−0.0254 (14)
N10	0.054 (2)	0.061 (2)	0.084 (2)	−0.0211 (17)	−0.0054 (19)	−0.038 (2)
N11	0.0400 (16)	0.0351 (15)	0.0536 (17)	−0.0051 (12)	−0.0088 (13)	−0.0188 (13)
N12	0.0365 (16)	0.0554 (19)	0.0568 (18)	−0.0042 (14)	−0.0047 (14)	−0.0313 (16)
C27	0.250 (10)	0.067 (4)	0.335 (13)	0.000 (5)	−0.219 (10)	−0.048 (6)
O1	0.139 (3)	0.066 (2)	0.123 (3)	0.013 (2)	−0.044 (3)	−0.051 (2)
O1W	0.0514 (16)	0.0555 (16)	0.0523 (15)	0.0069 (12)	−0.0001 (12)	−0.0199 (13)
O2W	0.119 (3)	0.068 (2)	0.0630 (19)	−0.0367 (19)	−0.0044 (18)	−0.0249 (16)

### Geometric parameters (Å, °)

**Table:**

Zn1—N7	2.096 (3)	C16—H16A	0.9300
Zn1—N2	2.103 (2)	C17—N6	1.343 (4)
Zn1—N11	2.133 (3)	C17—C18	1.473 (4)
Zn1—N12	2.148 (3)	C18—N7	1.329 (4)
Zn1—N1	2.269 (3)	C18—N9	1.341 (4)
Zn1—N6	2.293 (3)	C19—N8	1.340 (4)
C1—N1	1.334 (4)	C19—N9	1.347 (4)
C1—C2	1.370 (5)	C19—C20	1.470 (4)
C1—H1A	0.9300	C20—C24	1.379 (5)
C2—C3	1.376 (6)	C20—C21	1.381 (5)
C2—H2A	0.9300	C21—C22	1.382 (5)
C3—C4	1.379 (5)	C21—H21A	0.9300
C3—H3A	0.9300	C22—N10	1.324 (5)
C4—C5	1.374 (5)	C22—H22A	0.9300
C4—H4A	0.9300	C23—N10	1.325 (5)
C5—N1	1.347 (4)	C23—C24	1.377 (5)
C5—C6	1.465 (4)	C23—H23A	0.9300
C6—N2	1.324 (4)	C24—H24A	0.9300
C6—N4	1.347 (4)	C25—N11	1.470 (4)
C7—N3	1.335 (4)	C25—C26	1.507 (5)
C7—N4	1.348 (4)	C25—H25A	0.9700
C7—C8	1.475 (4)	C25—H25B	0.9700
C8—C9	1.374 (5)	C26—N12	1.468 (5)
C8—C12	1.387 (5)	C26—H26A	0.9700
C9—C10	1.380 (5)	C26—H26B	0.9700
C9—H9A	0.9300	N2—N3	1.364 (3)
C10—N5	1.335 (5)	N7—N8	1.366 (4)
C10—H10A	0.9300	N11—H11B	0.8999
C11—N5	1.330 (5)	N11—H11C	0.9001
C11—C12	1.374 (5)	N12—H12B	0.9006
C11—H11A	0.9300	N12—H12C	0.8999
C12—H12A	0.9300	C27—O1	1.485 (8)
C13—N6	1.330 (4)	C27—H27A	0.9600
C13—C14	1.361 (5)	C27—H27B	0.9600
C13—H13A	0.9300	C27—H27C	0.9600
C14—C15	1.372 (5)	O1—H1B	0.9002
C14—H14A	0.9300	O1W—H1WA	0.8500
C15—C16	1.379 (5)	O1W—H1WB	0.8506
C15—H15A	0.9300	O2W—H2WA	0.8501
C16—C17	1.379 (5)	O2W—H2WB	0.8506
			
N7—Zn1—N2	156.47 (11)	N8—C19—N9	114.0 (3)
N7—Zn1—N11	99.48 (10)	N8—C19—C20	123.6 (3)
N2—Zn1—N11	98.05 (10)	N9—C19—C20	122.4 (3)
N7—Zn1—N12	101.58 (10)	C24—C20—C21	116.9 (3)
N2—Zn1—N12	96.28 (11)	C24—C20—C19	121.0 (3)
N11—Zn1—N12	81.99 (11)	C21—C20—C19	122.2 (3)
N7—Zn1—N1	89.05 (10)	C20—C21—C22	119.0 (3)
N2—Zn1—N1	74.90 (10)	C20—C21—H21A	120.5
N11—Zn1—N1	170.61 (10)	C22—C21—H21A	120.5
N12—Zn1—N1	92.50 (11)	N10—C22—C21	124.5 (4)
N7—Zn1—N6	75.35 (10)	N10—C22—H22A	117.7
N2—Zn1—N6	88.45 (10)	C21—C22—H22A	117.7
N11—Zn1—N6	92.21 (10)	N10—C23—C24	124.0 (4)
N12—Zn1—N6	172.96 (10)	N10—C23—H23A	118.0
N1—Zn1—N6	93.77 (10)	C24—C23—H23A	118.0
N1—C1—C2	123.1 (3)	C23—C24—C20	119.8 (4)
N1—C1—H1A	118.5	C23—C24—H24A	120.1
C2—C1—H1A	118.5	C20—C24—H24A	120.1
C1—C2—C3	118.7 (3)	N11—C25—C26	109.3 (3)
C1—C2—H2A	120.6	N11—C25—H25A	109.8
C3—C2—H2A	120.6	C26—C25—H25A	109.8
C2—C3—C4	118.9 (4)	N11—C25—H25B	109.8
C2—C3—H3A	120.5	C26—C25—H25B	109.8
C4—C3—H3A	120.5	H25A—C25—H25B	108.3
C5—C4—C3	119.2 (3)	N12—C26—C25	108.8 (3)
C5—C4—H4A	120.4	N12—C26—H26A	109.9
C3—C4—H4A	120.4	C25—C26—H26A	109.9
N1—C5—C4	122.0 (3)	N12—C26—H26B	109.9
N1—C5—C6	113.5 (3)	C25—C26—H26B	109.9
C4—C5—C6	124.5 (3)	H26A—C26—H26B	108.3
N2—C6—N4	113.5 (3)	C1—N1—C5	118.1 (3)
N2—C6—C5	119.7 (3)	C1—N1—Zn1	127.8 (2)
N4—C6—C5	126.8 (3)	C5—N1—Zn1	114.1 (2)
N3—C7—N4	114.2 (3)	C6—N2—N3	106.6 (2)
N3—C7—C8	121.6 (3)	C6—N2—Zn1	117.1 (2)
N4—C7—C8	124.1 (3)	N3—N2—Zn1	136.0 (2)
C9—C8—C12	117.5 (3)	C7—N3—N2	104.7 (3)
C9—C8—C7	121.1 (3)	C6—N4—C7	101.1 (3)
C12—C8—C7	121.4 (3)	C11—N5—C10	115.7 (3)
C8—C9—C10	119.3 (3)	C13—N6—C17	117.3 (3)
C8—C9—H9A	120.4	C13—N6—Zn1	129.4 (2)
C10—C9—H9A	120.4	C17—N6—Zn1	113.2 (2)
N5—C10—C9	124.1 (4)	C18—N7—N8	106.3 (2)
N5—C10—H10A	117.9	C18—N7—Zn1	117.0 (2)
C9—C10—H10A	117.9	N8—N7—Zn1	136.7 (2)
N5—C11—C12	124.6 (4)	C19—N8—N7	104.7 (2)
N5—C11—H11A	117.7	C18—N9—C19	101.3 (3)
C12—C11—H11A	117.7	C22—N10—C23	115.9 (3)
C11—C12—C8	118.9 (4)	C25—N11—Zn1	107.1 (2)
C11—C12—H12A	120.6	C25—N11—H11B	99.2
C8—C12—H12A	120.6	Zn1—N11—H11B	112.6
N6—C13—C14	123.6 (4)	C25—N11—H11C	97.5
N6—C13—H13A	118.2	Zn1—N11—H11C	113.1
C14—C13—H13A	118.2	H11B—N11—H11C	123.5
C13—C14—C15	119.1 (4)	C26—N12—Zn1	107.1 (2)
C13—C14—H14A	120.5	C26—N12—H12B	104.9
C15—C14—H14A	120.5	Zn1—N12—H12B	127.8
C14—C15—C16	118.8 (4)	C26—N12—H12C	110.1
C14—C15—H15A	120.6	Zn1—N12—H12C	107.7
C16—C15—H15A	120.6	H12B—N12—H12C	98.5
C17—C16—C15	118.5 (3)	O1—C27—H27A	109.5
C17—C16—H16A	120.7	O1—C27—H27B	109.5
C15—C16—H16A	120.7	H27A—C27—H27B	109.5
N6—C17—C16	122.7 (3)	O1—C27—H27C	109.5
N6—C17—C18	113.9 (3)	H27A—C27—H27C	109.5
C16—C17—C18	123.4 (3)	H27B—C27—H27C	109.5
N7—C18—N9	113.8 (3)	C27—O1—H1B	112.0
N7—C18—C17	120.1 (3)	H1WA—O1W—H1WB	104.0
N9—C18—C17	126.0 (3)	H2WA—O2W—H2WB	133.0

### Hydrogen-bond geometry (Å, °)

**Table:**

*D*—H···*A*	*D*—H	H···*A*	*D*···*A*	*D*—H···*A*
N11—H11B···O1W^i^	0.90	2.27	3.166 (4)	172
N11—H11C···O2W^ii^	0.90	2.17	3.034 (4)	161
N12—H12B···N5^iii^	0.90	2.54	3.321 (4)	146
N12—H12C···O1W	0.90	2.45	3.331 (4)	165
O1—H1B···N4^iv^	0.90	2.02	2.897 (4)	163
O1W—H1WA···N3^v^	0.85	2.04	2.887 (4)	171
O1W—H1WB···N8	0.85	2.09	2.859 (4)	150
O2W—H2WA···O1	0.85	1.94	2.778 (4)	169
O2W—H2WB···O1W	0.85	2.00	2.787 (4)	153

Symmetry codes: (i) *x*+1, *y*, *z*; (ii) −*x*+1, −*y*+1, −*z*+1; (iii) −*x*+2, −*y*+2, −*z*+1; (iv) −*x*+1, −*y*+2, −*z*+1; (v) *x*−1, *y*, *z*.
